# Complications following equine sacroiliac region analgesia are uncommon: A study in 118 horses

**DOI:** 10.1371/journal.pone.0247781

**Published:** 2021-03-02

**Authors:** Samuel C. J. Offord, Rachel M. Read, Camilla J. Pudney, Andrew P. Bathe

**Affiliations:** Rossdales Equine Hospital and Diagnostic Centre, Exning, Newmarket, Suffolk, United Kingdom; Universita degli Studi di Padova, ITALY

## Abstract

**Background:**

Diagnosis of sacroiliac region pain is supported by a positive response to sacroiliac region analgesia (SIRA). Varying techniques have been described for SIRA; with clinician preference often dictating method. Potential complications following SIRA include ataxia and recumbency. No study has specifically evaluated the prevalence of complications.

**Objectives:**

To describe the complication prevalence following SIRA in a referral clinic.

**Study design:**

Retrospective cohort study.

**Methods:**

Review of records from horses presented to two of the authors at Rossdales, Newmarket, between January 2014 and December 2018, that underwent SIRA. Injection was performed using a blind midline approach with 20 mL mepivacaine (Intra-Epicaine 20mg/ml; Dechra) infiltrated through a straight 18 gauge 8.9cm spinal needle subdivided into four sub-locations per block.

**Results:**

118 horses were included, with 167 individual blocks. One horse showed a mild hindlimb gait abnormality following SIRA, which resolved uneventfully over 3 hours; complication rate 1/118 horses (0.85%; 95% CI: 0,2.5%), 1/167 joints (0.60%; 95% CI: 0,1.8%). SIRA subjectively improved lameness/performance in 132/167 (79%) joints. 49/118 (42%) received bilateral SIRA with 53/118 (45%) evaluated ridden following SIRA.

**Main limitations:**

Small population numbers with low complication prevalence rate.

**Conclusions:**

SIRA, using the described technique, has a low (0.85%) prevalence of complications.

## Introduction

Sacroiliac region pain is a well-reported finding in horses presenting with lameness or poor performance [1–4]. Various non-specific provocation techniques are described to identify sacroiliac region pain [5]. The sacroiliac region is not easily evaluated with diagnostic imaging [6–9] and bone-phase gamma scintigraphy is not recommended in isolation for a diagnosis of sacroiliac joint dysfunction due to the variability in radiopharmaceutical uptake in this region [1]. Therefore, sacroiliac region pain can only be confirmed by a positive response to either diagnostic local analgesia or infiltration of medication [1, 2, 10]. Sacroiliac region pain is commonly seen in combination with other sites of lameness [1, 2]. A previous study by Barstow and Dyson [1] identified hindlimb lameness in 80% of horses which was not caused by but in combination with sacroiliac joint region pain; with 89% of these horses diagnosed with proximal suspensory desmitis [1].

Sacroiliac regional analgesia (SIRA) has previously been reported in detail, with multiple techniques described, both blind and ultrasound guided, and the majority reporting regional rather than intra-articular blocks [11–14]. Diagnosis of true sacroiliac joint pain may be difficult to differentiate from more generalised lumbosacral pain, with technique and volume of local anaesthetic used likely important factors [11].

Possible complications following SIRA include: ataxia, recumbency, infection, puncture of major vessels and rectum [12, 13]. Local anaesthetic diffusion to nerves, the lumbosacral region and the sacrocaudalis dorsalis muscle body is possible [10, 15]. Placement of the needle under ultrasonographic guidance has been advocated for reducing the risk of inadvertent injection into or around the vertebral canal and sciatic nerve [12]. The caudal approach is not recommended for diagnostic analgesia due to the increased risk of diffusion of local anaesthetic around the sciatic nerve [12], with ultrasound guidance advocated to reduce the risk of injection into the rectum [12]. This technique involves placement of the needle 3-4cm caudal to the tuber sacrale in a caudodorsal-cranioventral direction and using ultrasound guidance to redirect as required to the sacroiliac joint, under the caudal aspect of the ilial wing [12]. Due to anecdotal concerns regarding complications following SIRA there can be a reluctance to perform this block. A recent international survey with veterinary surgeons invited to participate reported 15 horses that became recumbent following SIRA, with 14 of these making a full recovery [10].

The aim of this study was to report the prevalence of complications following SIRA using a blind midline technique in one referral clinic. To the authors knowledge no study has specifically reported the prevalence of complications following SIRA. A secondary objective to assist clinicians in this decision process was to provide data on the proportion of horses improved using the described SIRA technique. Quantitative data is required to make an informed decision regarding the safety of infiltration of local anaesthetic into the sacroiliac region, which can then be weighed against the potential benefits of SIRA.

## Materials & methods

Ethic animal research statement: This was a retrospective study of clinical records. Explicit informed owner consent for inclusion not given. Written consent for use of clinical records in scientific studies and research is given by each owner when signing the consent form at the time of admission to Rossdales Diagnostic Centre.

### Population

Medical records of horses admitted to Rossdales Diagnostic Centre, Newmarket, UK from 1^st^ January 2014 to 31^st^ December 2018, under the care of the two senior authors (APB & RR), that underwent SIRA were included. Horses were excluded from this study if the local anaesthetic used was combined with any other product, including corticosteroid, at the time of SIRA being performed, or if the horse underwent repeat sacroiliac region injection for the purpose of infiltration of medication at the same appointment as SIRA was performed. Horses with complete clinical records including both diagnostic and treatment data were collated.

Data collated from clinical records included age, breed, gender; with classification of horse use into dressage, show jumping, eventing and general-purpose categories. All sites of diagnostic local analgesia, the block responses, the sedation requirement and whether a ridden evaluation was performed were noted. Complications following SIRA whilst the horse was on site were recorded. A retrospective review of clinical records identified horses that had been re-evaluated at the clinic. If they were not re-examined a telephone follow-up was performed, with any client reported complications noted.

### Examination

All horses underwent a thorough orthopaedic focussed clinical examination, including palpation over the pelvis and sacroiliac region. All horses were evaluated dynamically in-hand in a straight line on a firm surface. Lunging was performed on a 15m circle on both hard (tarmac) and soft (waxed sand and rubber) surfaces in trot, with the horse also cantered on the soft surface. Ridden evaluation, when clinically required, was performed in a purpose-built arena (waxed sand and rubber) with a combination of riders depending on the situation including; the usual rider, riders’ trainer or a trained employee rider of the clinic. Lameness was graded on a scale 0–10 [16], a quantitative gait analysis system was not used. An equal to or greater than 50% improvement in lameness or way of going under saddle was recorded as a positive response to SIRA.

### Sacroiliac region analgesia procedure

The horse was controlled using a headcollar and bridle, with the majority of horses held by a professional veterinary technician and the remainder held by the owner or rider. A nose or neck twitch was used on a case-by-case basis depending on the temperament of the horse, with sedation only used if essential. All SIRA was performed in stocks and the sacroiliac region aseptically prepared, with the area clipped if the horse had a thick coat. The tail was wrapped with cohesive bandaging material to prevent contamination of the injection site.

**Fig 1:**
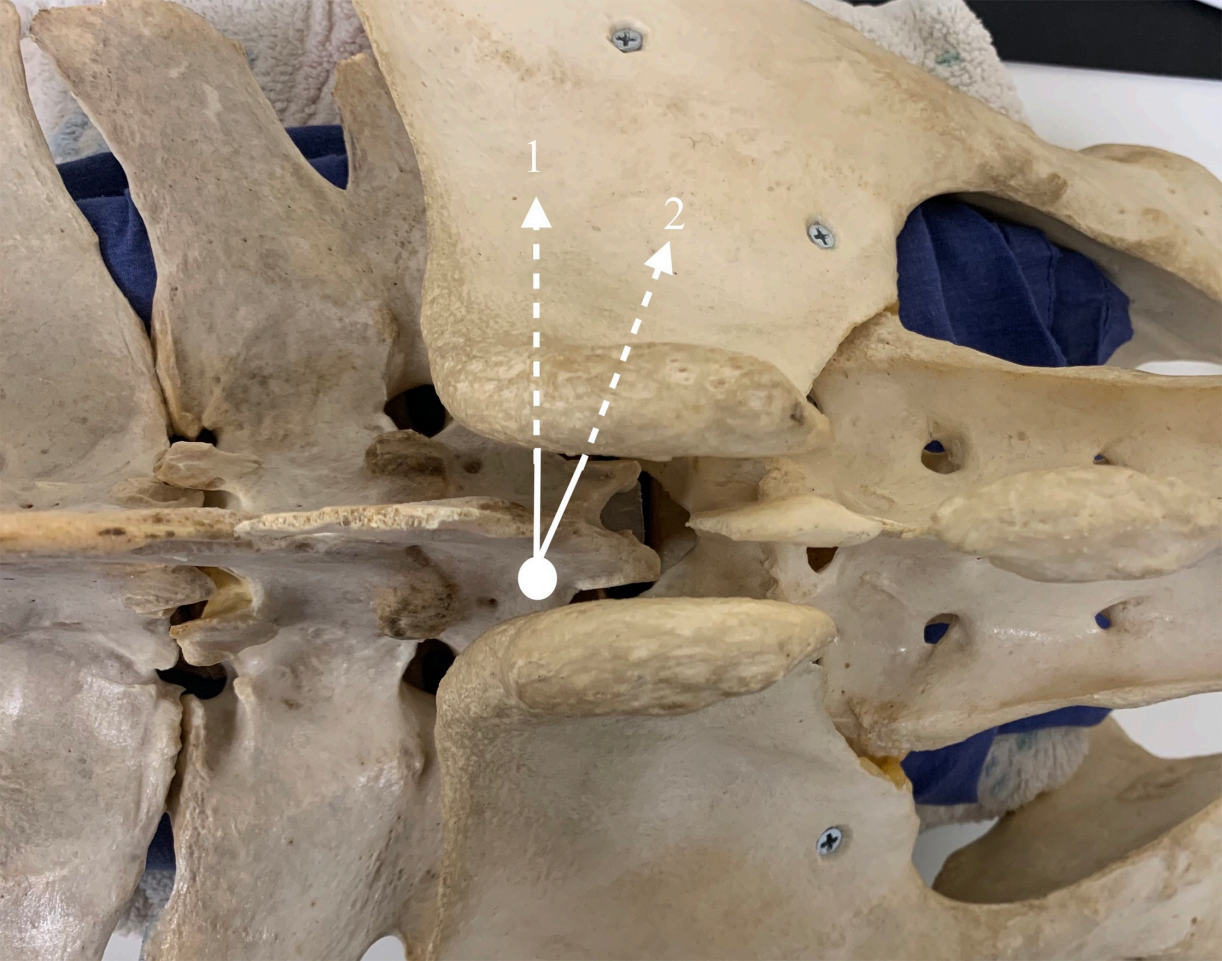
Depiction of sacroiliac region analgesia (SIRA) procedure. Dorsal view over a pelvic bone specimen with tuber sacrale at the centre of the image. Arrows 1 and 2 indicate the needle direction and the order for injection of local anaesthetic for right SIRA. The white circle indicates the site of needle insertion through the skin, with the dotted lines representing the needle path ventral to the iliac wing.

A clean, non-sterile, blind injection using a midline approach was employed (Fig 1), using a modified contralateral medial approach similar to that described by *Barstow et al* [1] and *Engeli et al* [14]. A total of 20ml of 2% mepivicaine hydrochloride (Intra-Epicaine 20mg/ml; Dechra) was used per block, infiltrated through a straight 18g 8.9cm spinal needle, and subdivided equally into 4 locations. The cranial aspect of each tuber sacrale was palpated and the needle inserted axial to the cranial aspect of the contralateral tuber sacrale, aiming perpendicular to the tuber sacrale and toward the caudal aspect of the ipsilateral tuber coxae. The needle was advanced dorsal to and between the diverging dorsal spinous processes of the sixth lumbar and first sacral vertebrae and ventral to the tuber sacrale up to the hub of the needle, with the angle of the needle dependent on horse size and the width of space between the tuber sacrale. The following four locations for deposition of local anaesthetic were used through one skin puncture site: 5mls deep and then the needle is withdrawn 0.5-1cm to deposit a further 5mls; the needle was then redirected caudad approximately 20^O^ with 5mls deep and 5mls with the needle withdrawn as previously described. This technique was used for all of the joints blocked by all of the authors. Horses received either unilateral or bilateral SIRA, as clinically indicated. If bilateral both blocks were either performed simultaneously or sequentially (one side blocked; horse lunged after 10 minutes; and then the other side blocked). Each horse was re-examined ten minutes following SIRA, with any complications noted at this stage and subsequently recorded.

### Statistical methods

A sample size calculation [17] was performed, with a 95% confidence interval and 1% of the population affected, based on extracted data from a previous study [1] a sample size of 83 was required for detection of a 1% complication prevalence rate. Prevalence of complications following SIRA was calculated as a percentage for both the number of horses and joints affected. Confidence intervals were calculated using a normal approximation to the binomial calculation [18], with the confidence level set at 95%.

## Results

A total of 118 horses satisfied the inclusion criteria and consisted of 88 geldings, 29 mares and one stallion. There were a wide range of breeds, with warmblood horses predominating (43/118), with this study also including the following breed types: Andalucian, Cob, Connemara, Irish Draught, Thoroughbred and Thoroughbred cross, Trackehner, New Forest and Welsh Section D. The study population consisted predominantly of sport horses, with some general-purpose animals; the use of the horse was representative of the clinical caseload presented to the authors. Horses presented with a variety of clinical signs; including poor performance, lameness and behavioural concerns.

A total of 118 horses underwent SIRA with 167 individual sacroiliac regions blocked. Forty-nine horses (42%) received bilateral regional analgesia, with 41 sequential and 8 simultaneous bilateral blocks.

One joint (1/167; 0.60%; 95% CI: 0,1.8%) in one horse (1/118; 0.85%; 95% CI: 0,2.5%), a 9-year-old British Warmblood gelding in good body condition score that underwent left SIRA, encountered a complication as a result of the SIRA procedure. The horse was re-examined 10 minutes following placement of the block, at which point a mild unilateral left hindlimb gait abnormality was noted. The horse knocked into itself and was unable to be dynamically re-evaluated. No treatment was required, with the horse recovering uneventfully after three hours.

Follow-up of each horse was analysed with a large proportion of horses (95/118) re-evaluated at the clinic at variable time points following the examination when the horse underwent SIRA. The remaining 19 horses not re-evaluated at the clinic, were followed up by client telephone conversation, with no client reporting any complications as a result of SIRA. Four horses presented with severe behavioural issues endangering human safety and were subsequently euthanised at the same appointment as SIRA was performed. All four horses improved to the block, but longer term follow up cannot be reported for these.

A 79% positive block rate was achieved for both individual horse and individual blocks, with 132/167 blocks improving lameness or way of going under saddle in 93/118 horses as assessed by the attending clinician. Fifty-three horses (45%) were assessed ridden following the block/s, with no complications encountered throughout the ridden evaluation. During the ridden evaluation rider feedback on horse performance was requested, with 39/53 (74%) riders reporting an improvement.

Four horses required sedation for the block to be placed; this was based on clinical judgment of the horses’ temperament. Intravenous sedation used varied depending on clinical indication, with three horses receiving xylazine (Virbaxyl 10%, Virbac; dose range 0.14–0.4 mg/kg) and one horse romifidine (Sedivet, Boehringer Ingelheim; dose 0.01 mg/kg). All horses that were sedated prior to SIRA were successfully re-evaluated following placement of the block, with no issues in this population due to the use of sedation.

Adjunctive sites of pain were diagnosed in 98/118 (83%) horses: hindlimb proximal suspensory pain in 72 horses (61%) and 28 horses (24%) shown to have stifle pain. Combined hindlimb proximal suspensory ligament and stifle joint pain was found in 8/118 horses (7%). Other sites of pain were identified in 19 horses, with some improving to multiple sites including the hindlimb proximal suspensory ligament and stifle. Diagnostic local analgesia distal to the sacroiliac region failed to improve lameness and/or performance in 20/118 horses, with SIRA improving 17 of these, identifying sacroiliac region pain as the sole cause of lameness or poor performance in this subset of horses. Lameness grades have not been reported, due to the frequently multifactorial cause of the lameness, as reported above, which would not solely represent sacroiliac region pain.

## Discussion

This study reports the prevalence of complications as 0.85% following SIRA using a blind midline approach in horses. Therefore, from this data it can be concluded that complications following SIRA are uncommon using this technique. This statement is based on the National Institute for Health and Care Excellence (NICE) guidelines for drug side effects, which quantify uncommon side effects as a rate between 0.1% and 1% [19].

A previous study into sacroiliac region pain1], which focussed on the clinical features and diagnosis of sacroiliac joint region pain, reported 2/284 horses to be ataxic following sacroiliac region analgesia, with a further two horses removed from the study due to ataxia and an inability to evaluate the block. The complication rate of 0.70% per horse (2/284) correlates well with the prevalence identified in this study; however, the main difference between this study and the current data is that a different SIRA technique was used [1]. A recent study [10] reported a within-clinic recumbency rate of approximately 0.3% (3 out of approximately 1180) following SIRA, with the authors estimating 1–2% of horses showed mild ataxia that precluded ridden re-evaluation [10].

Ataxia and/or recumbency may be induced following SIRA by inadvertent deposition of local anaesthetic into the epidural space, whilst an abnormal gait will be appreciated if there is perineural injection of hindlimb nerves [5, 10, 12]. The greater sciatic foramen, located ventromedial to the sacroiliac joint, allows passage of the sciatic nerve and cranial gluteal nerve [5, 15], and is therefore close to the local anaesthetic deposition site for SIRA. If the sciatic nerve is infiltrated both sensory and motor deficits will be appreciated [20]. This feature may reduce the risk of false positive results following SIRA. If there is inadvertent unilateral infiltration of the nerve during SIRA the horse will remain standing as long as the contralateral limb remains functional. However, ataxia of both hindlimbs and/or recumbency would be caused in cases of injection into the epidural space.

Ataxia and recumbency are the most frequently reported complications and are reported to be quick in onset following SIRA, with the majority of horses noted to be ataxic or become recumbent within 20 minutes [1, 10]. The one horse that displayed a mild gait abnormality in this study was identified when re-evaluated ten minutes following the block, which correlates with the previous reports. The reported duration of action of mepivacaine for nerve blocks is 2–3 hours [21] with this horse taking 3 hours to completely recover from the ataxia. No insidious onset complications were identified in this study through either a repeat veterinary examination or a follow-up telephone conversation which, although not as thorough as a veterinary examination, does give long-term information following SIRA not previously reported.

The volume of local anaesthetic, the technique including needle type and length and whether it is performed blind or ultrasound guided will likely influence the rate of complications and the areas desensitised; however, no study has specifically investigated the influence of these factors. Knowledge of the structures blocked by each SIRA technique may help differentiate between true sacroiliac joint and more generalised lumbosacral region pain [11–14, 22]. The anatomical structures contributing to sacroiliac region pain have been discussed, but currently the contribution of each of these is unknown [1, 11]. Currently clinician experience and preference appear to be the main impacts on SIRA method.

Ultrasound guidance was not used in this study as easily palpable landmarks were available for orientation of the operator. Use of ultrasound guidance has been advocated to increase accuracy of injections; however, the caudal ultrasound guided approach is not recommended for diagnostic analgesia [12]. When the cranial or midline approaches are used ultrasonographic assistance only ensures placement of the needle ventral to the ilial wing; with the needle bevel obscured by bone and further needle advancement performed blind [11–13, 22].

A variety of techniques, needle lengths, needle curvature and volumes of local anaesthetic for SIRA are reported, with no one method described as superior [11, 22]. The medial contralateral approach used in this study is most similar to the cranial or medial contralateral approach previously described [1, 10, 14]. The cranial contralateral technique was used in the largest case series of sacroiliac region pain [1]. The needle entry point for the cranial contralateral technique described by *Barstow & Dyson* [1] is further from the sacroiliac joint, oblique in three planes and used a longer needle, which may potentially increase the technical difficulty and reduce accuracy. Our technique uses a shorter needle, with needle placement only in two planes initially (Fig 1), which will likely make this SIRA technique easier for the operator.

For SIRA the authors use a 3.5” (8.9cm) straight spinal needle, as described in this study. A 20-25cm length needle is advocated by some authors for peri-articular sacroiliac joint injections in medium and large breed horses, based on the reported distance from the skin to the sacroiliac joint [11, 14]. A longer needle is a possible factor that may contribute to recumbency following SIRA and would potentially deposit local anaesthetic closer to the greater sciatic foramen. In contrast a shorter needle may reduce the proximity to the sacroiliac joint and allow wider diffusion of local anaesthetic to the lumbosacral region [11]. Curved needles are reportedly beneficial to follow the ventral contour of the ilial wing and reduce the distance between the needle tip and the sacroiliac joint; an estimated 40^o^ manual contour of a 20-25cm needle is described [11]. However, in the authors experience accurate placement of the tip of a long, curved needle is far more technically challenging and therefore risks inaccurate deposition of local anaesthetic.

Varying volumes of local anaesthetic have been described for SIRA from 6-20mls [2, 23], with these two similarly using the same concentration of mepivacaine as the current study. Widespread diffusion has been reported with larger volumes [11] potentially desensitising regional lumbosacral pain rather than sacroiliac joint pain. Larger volumes, given the wider diffusion, may increase the risk for iatrogenic side effects, particularly abnormal gaits, but this will likely also be dependent on technique and needle length. *Barstow & Dyson* [1] have used 12-15ml of mepivacaine, in comparison to our study using 20ml mepivacaine with no major complications.

In our study the majority of horses that underwent bilateral SIRA were blocked sequentially; 8 horses underwent simultaneous SIRA with no complications encountered as a result of this. Bilateral SIRA was performed in 13/15 horses that became recumbent [10], which we presume was simultaneous. It is thought by the authors, but not statistically proven, that sequential SIRA may be a lower risk compared to simultaneous as previously discussed. However, further data analysing sequential and simultaneous SIRA, ideally with a control group is required to support this statement.

This study showed an improvement in lameness and way of going under saddle in 79% of the population following SIRA, indicating that a significant portion of pain would not have been identified if SIRA hadn’t been performed. A proportion (20/118) of horses did not improve to diagnostic local anaesthesia in the pelvic limb/s, with 17/20 improved following SIRA. The lameness or poor performance would have been undiagnosed if SIRA hadn’t been performed in these cases.

Diagnostic local anaesthesia of the pelvic limb/s was used in the majority (110/118) of cases prior to SIRA, with adjunctive sites of pain often identified. Proximal suspensory ligament and stifle joint pain were most frequently identified in 61% and 24% of the population respectively. These data confirm the necessity to consider other causes of lameness in combination with sacroiliac region pain.

Our study used a professional handler to restrain the majority of horses, with all horses placed into stocks, which minimises patient movement, our requirement for sedation and improves operator safety. Interestingly, the recent survey of recumbency following SIRA [10] showed that 13/15 horses were restrained in stocks, which is likely a representation of the proportion of veterinary surgeons who use stocks for SIRA. A small proportion of horses in this study (4/118) required sedation for placement of the block, in comparison to 7/15 horses administered sedation, prior to SIRA being performed, in the case series of recumbency following SIRA [10]. The effect of sedation on ataxia and recumbency following SIRA is unclear but logically sedation would reduce patient movement and increase accuracy of needle placement, with the dosages of sedation reported unlikely to influence any ataxia or recumbency.

In this study no complications were associated with a ridden assessment following SIRA, with 45% of the study population assessed under saddle. A perceived improvement by the rider was found in 39/53 (74%) cases which strengthens the use a ridden examination following SIRA, with the added benefit of rider feedback on the horses’ movement.

### Study limitations

The complication rate was low, which may be influenced by the population size analysed. A sample size calculation was performed prior to data collection to reduce the risk of a low powered study. The same operator did not perform all of the blocks; however, the same approach was used for all regional analgesia performed. The technique was taught and supervised by APB, ensuring continuity and repeatability whilst also demonstrating that this technique can easily be performed by multiple operators. Analysis of lameness was performed by the two experienced senior authors (APB and RR), with no quantitative gait analysis system used.

## Conclusions

This study has shown that complications following SIRA are uncommon, using a blind midline technique. A large proportion of the population (79%) improved following this block, with this site of pain unidentified if SIRA had not been performed. The low prevalence of complications and high proportion of improvement to SIRA identified in this study demonstrate that the benefits of diagnosing sacroiliac region pain outweigh the potential risk of complications following SIRA.

## Supporting information

S1 Table Minimum dataset detailing horse information, block responses and record of complications. (XLSX)
